# Considering the Role of Human Empathy in AI-Driven Therapy

**DOI:** 10.2196/56529

**Published:** 2024-06-11

**Authors:** Matan Rubin, Hadar Arnon, Jonathan D Huppert, Anat Perry

**Affiliations:** 1 Psychology Department Hebrew University of Jerusalem Jerusalem Israel

**Keywords:** empathy, empathetic, empathic, artificial empathy, AI, artificial intelligence, mental health, machine learning, algorithm, algorithms, predictive model, predictive models, predictive analytics, predictive system, practical model, practical models, model, models, therapy, mental illness, mental illnesses, mental disease, mental diseases, mood disorder, mood disorders, emotion, emotions, e-mental health, digital mental health, internet-based therapy

## Abstract

Recent breakthroughs in artificial intelligence (AI) language models have elevated the vision of using conversational AI support for mental health, with a growing body of literature indicating varying degrees of efficacy. In this paper, we ask when, in therapy, it will be easier to replace humans and, conversely, in what instances, human connection will still be more valued. We suggest that empathy lies at the heart of the answer to this question. First, we define different aspects of empathy and outline the potential empathic capabilities of humans versus AI. Next, we consider what determines when these aspects are needed most in therapy, both from the perspective of therapeutic methodology and from the perspective of patient objectives. Ultimately, our goal is to prompt further investigation and dialogue, urging both practitioners and scholars engaged in AI-mediated therapy to keep these questions and considerations in mind when investigating AI implementation in mental health.

## Introduction

The prospect of using machine learning algorithms for automated health care responses and counseling has long been considered [1]. Such algorithms could have vast benefits ranging from increased accessibility and affordability of mental health services, reduced waiting times, and personalized treatment options, to the potential to reach underserved populations and combat the escalating loneliness epidemic [2]. Recent breakthroughs in artificial intelligence (AI) language models have elevated this vision, as evidenced by a growing body of literature indicating varying degrees of efficacy. For instance, studies demonstrate that digital chatbots are proficient in delivering psychoeducation and improving treatment adherence over short durations [3]. Additionally, AI-driven chatbots have been effectively used to impart strategies derived from positive psychology and cognitive behavioral techniques to mitigate stress and enhance subjective well-being [4]. AI chatbots can also provide preliminary support in the absence of a therapist by prompting self-reflective questioning and facilitating emotion regulation in challenging scenarios [5,6]. Machine learning can also be used in order to detect symptom changes in new ways [7]. In the realm of medicine, a recent study revealed that the responses from GPT-3 received higher ratings for the quality of medical advice compared to physicians. Moreover, these responses were perceived to exhibit significantly more empathy compared to those from physicians [8].

While the potential for AI chatbots to take over certain elements of the therapeutic process exists, there are compelling reasons to believe that they cannot completely substitute for the human element. This raises a critical question: under what circumstances will human therapists remain indispensable, and conversely, when could they feasibly be replaced by AI models? We suggest that part of the answer may reside in an exploration of the role of empathy in the therapeutic process. In the following paper, we address the multifaceted nature of empathy, including its cognitive, emotional, and motivational aspects. We claim that in those cases where emotional or motivational empathy is needed, humans will be harder to replace. We then consider what determines when these aspects are needed most in therapy—whether certain therapeutic approaches, particular patient goals, or specific points within the therapeutic timeline. Our inquiry explores these considerations from both the perspective of therapeutic methods and patient objectives. Ultimately, our goal is to prompt further investigation and dialogue, urging both practitioners and scholars engaged in AI-mediated therapy to consider these issues through the lens of empathy and human connection.

## Empathy

A comprehensive definition of empathy recognizes 3 dimensions of empathic engagement: cognitive empathy, or mentalizing, which pertains to the recognition and understanding of the emotional states of others; emotional empathy, or affective sharing, which involves resonating with others’ emotional experiences while maintaining self-other differentiation; and motivational empathy, often termed empathic concern or compassion, which encompasses feelings of concern for another’s welfare and a readiness to act to enhance their well-being [9].

Current advances in natural language processing and facial recognition technologies have positioned AI-based algorithms at the forefront of discerning emotional states [10,11], with projections suggesting that they may reach or surpass human capability in the near future. Therefore, in the most basic sense of cognitive empathy as recognition of the other’s emotional state, AI algorithms will probably do quite well.

Nonetheless, AI, at least in its current form, does not exhibit the latter 2 empathic capacities. AI does not partake in emotional experiences—it neither shares in joy nor sorrow. Therefore, regardless of how eloquently it crafts a response to seem like it shares an emotional experience, this response will be untruthful, as it does not share any experience. While such responses may still have some benefits, they will probably not be experienced by the listener in the same way [12,13].

Moreover, conversational AI does not have the capacity to manifest genuine care and concern. Human expressions of empathic care signal a willingness to bear an emotional burden and expend limited cognitive-emotional resources on the interaction. Empathy, being potentially taxing, is selectively directed, often preferentially toward close relations and in group members, rather than those more distant [14]. In this way, such expressions signify the recipient’s importance and closeness to the empathizer. Indeed, studies show that, stripped of context and motivation, individuals often tend to avoid empathy [15,16]. Thus, whether in therapy or in social or professional realms, authentic expressions of empathy are significant to the recipient because they reflect a conscious commitment of time, thought, and emotional labor from the empathizer. Though these resources are inherently scarce for humans, they are unlimited for a conversational AI model. Its response is essentially cost-free, and it would react with comparable enthusiasm to anyone else. As a result, the conversational AI’s empathy fails to convey authentic care or indicate that the recipient holds any unique importance [17].

Empathy, and specifically its emotional and motivational components, has been consistently linked to positive outcomes in treatment. The extent of empathy expressed by the therapist and perceived by the patient has a substantial correlation with the success of the treatment [18]. Rogers [19] even describes the therapeutic process as a mutual participation in an emotional exchange, which is then accurately interpreted and reflected upon with the patient to facilitate understanding of their experiences. As Rogers articulated, comprehending the patient’s emotions (cognitive empathy) is imperative for endorsing and designing goals and interventions that confront these emotions. This process is underpinned by a commitment to assist and support the patient (motivational empathy), both of which stem from participating in the patient’s emotional journey (affective empathy).

Upon considering the importance of empathy for the therapeutic process and outcomes, as well as the limitations of AI discussed earlier, several questions arise. First, given the limitations, for which aspects of therapy could AI completely replace human therapists? Second, in aspects of therapy where human empathy is essential, how can AI algorithms assist therapists? For example, could it aid therapists in being more accurate or in being more committed to their patients (perhaps by enhancing therapist understanding and empathy and potentially reducing burnout)?

While we do not claim to give clear-cut answers, this paper explores these questions through dual lenses: the perspective of the therapeutic approach and the perspective that prioritizes specific motivations and needs of the patient.

## Perspectives in Psychotherapy

Psychotherapy encompasses a diverse spectrum of approaches. A major debate in the field of psychotherapy concerns the mechanism of change. To state it simplistically, one extreme viewpoint contends that the therapeutic relationship is the main mechanism [20], whereas the other extreme argues that therapeutic techniques or procedures are the main mechanism [21]. At times, these 2 stances are reflected in therapeutic approaches, such that psychodynamic (ie, Neo-Freudian) approaches tend to emphasize the therapeutic relationship whereas cognitive behavioral approaches tend to emphasize technique. In reality, most psychotherapies attempt to integrate some combination of the 2 and allow them to build on one another; there are techniques used to form the relationship, and relationships facilitate the use of techniques. Furthermore, a given act can be seen as both relationship-building and the administration of a technique. Essentially, the therapeutic process often demands that the therapist engage in a comparative analysis of emotional experiences with the patient, thus exercising some form of affective empathy, though it is possible that these affective components of empathy are more crucial in some therapeutic interventions than in others.

Despite the variance in therapeutic orientations, there is broad agreement that one of the fundamental transtheoretical elements critical for a successful outcome is the treatment’s working alliance [22]. Conventionally, the alliance is measured across 3 domains: agreement on therapeutic goals, agreement on the therapeutic activities needed to achieve these goals, and the warmth and genuineness of the connection. The working alliance is a significant predictor of treatment outcomes across all forms of psychotherapy [23,24]. Motivational empathy is intimately connected to a fruitful working alliance, particularly the aspect concerning warmth and care [25]. Such results were reported in early studies of cognitive behavioral therapy, where warmth was a predictor of symptom improvement [26], though subsequent findings have been equivocal [21].

One of the provocative findings regarding the alliance of client and therapist is that it appears to be similarly related to reported outcomes in face-to-face psychotherapy and in internet-based interventions (IBIs), which include asynchronous communications [24] with minimal therapist contact. Within the field of IBI, the presence of therapist support is predictive of more symptom improvement, less dropout, and greater adherence in comparison with unguided IBI [27,28] (though data are not conclusive [29]). Furthermore, the relationship with the internet-based program has been found to be predictive of symptom reduction, whereas the relationship with the therapist was predictive of adherence and dropout in a therapist-guided IBI [30]. These differences raise the possibility that although patients can potentially form a relationship with a digital interface, such a relationship differs in its benefits in comparison to a relationship with a therapist, and may not be able to create the same profound alliance and conversations with patients [31]. In other words, conversational AI and digital programs can be helpful in psychoeducation and administering practical techniques that alleviate symptoms, but they are unlikely to build a motivational relationship in the same way as a human therapist and client. Moreover, it has been claimed that even if conversational AI were to be used only for practical purposes alongside a human therapist, this may change and interact with the human dynamic in therapy, especially with the therapeutic alliance [32], which may possibly change how one experiences empathy throughout the therapeutic process.

## A Patient-Needs Perspective

**Figure 1:**
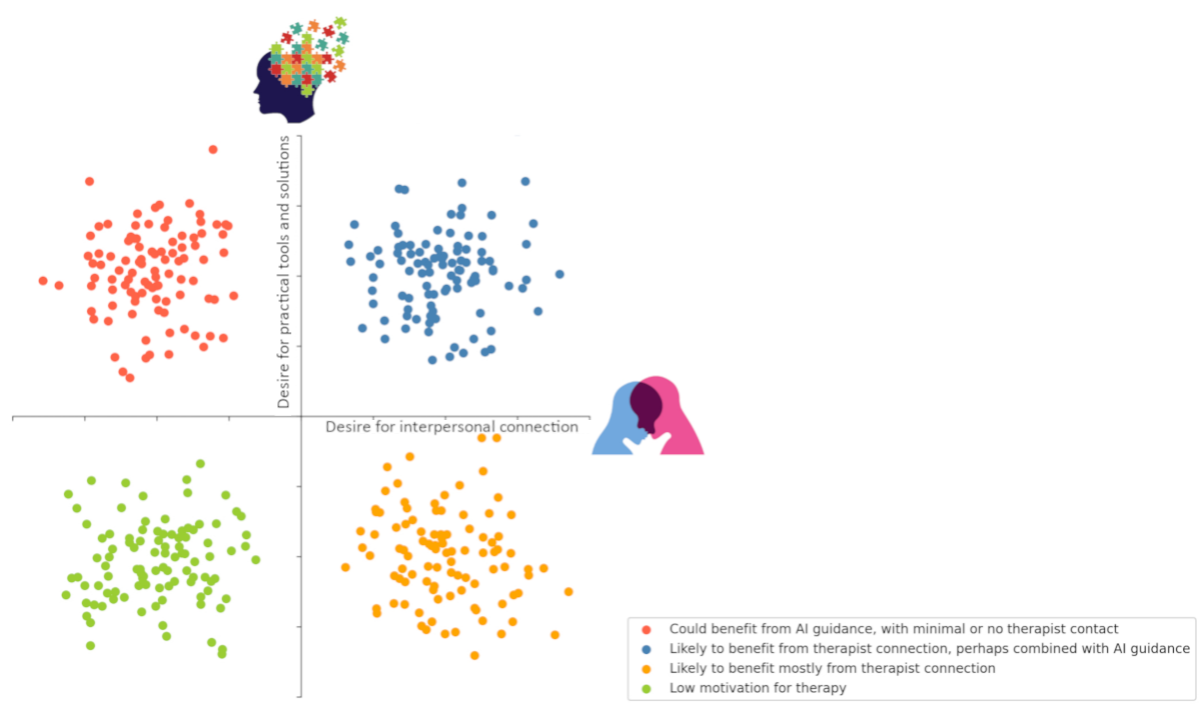
An illustration of different patients’ possible needs and their possible benefit from AI intervention or a human connection in therapy. AI: artificial intelligence.

The previous section examined the importance of empathy in therapy, from the perspective of therapeutic treatment approaches, which could be thought of as a continuum in terms of their theoretical mechanisms from skill acquisition (eg, cognitive behavioral therapy) to relationship-based (eg, interpersonal psychoanalysis). An alternate perspective considers a patient’s specific motivations and needs for seeking treatment, regardless of the therapist’s theoretical orientation. We contend that patients enter therapy with a variety of needs, motivations, and expectations, which can be conceptualized along two orthogonal continua including (1) a desire to acquire practical tools and (2) a desire for human connection and empathy. The relative emphasis on each dimension varies among patients according to their theory of change (ie, what they need to decrease distress or improve quality of life) and many other factors (such as their history of successful or unsuccessful treatments, stigma, culture, etc). In addition to individual variability, we contend that individuals can change in their theory and emphasis during therapy as a result of their cumulative experiences over the course of therapy. The more a patient seeks to acquire strategies to cope, the more conversational AI might be able to facilitate this process by providing psychoeducation, exercises, and the like. Conversely, the greater the patient’s need for human connection and empathy—be it for affirmation, a confidant for their thoughts and feelings, or simply the sense that someone cares—the less capable conversational AI might be in fulfilling these requirements (Figure 1). We note that although we see these as 2 dimensions, we would not expect many individuals who seek treatment to be low on both, as there would be no motivation to seek treatment (aside from appeasing others).

Adding to this complexity is the patient’s self-awareness and accuracy regarding their needs, which is often ambiguous (eg, they may believe they need practical tools when, in reality, they require empathic care more, or vice versa). It is conceivable that a patient might need coping skills for personal growth; yet, the most effective means of acquiring these tools could be through engagement with a compassionate therapist. Such a process may take time and require working on the therapeutic relationship, and needs may change during this period. We believe that when thinking of how to use AI systems in mental health contexts, we must not seek to use them as a “quick fix” solution to a specific problem at all times, as in some cases, a long-term human connection may be required to provide more thorough help.

Moreover, even in therapeutic interactions that are not complete courses of psychotherapy and are comprised of short, concrete interventions, such as crisis helplines, people still may need a human connection. Indeed, research shows that the main reason people call crisis helplines is to have someone to talk to [33]. While conversational AI systems can, at times, give the feeling of “being heard,” their responses have still been reported to have less value than those written by humans [13]. One could assume that repeat callers [34] to such helplines seek human connection and will not find it sufficient to only communicate with a bot. A bot-only approach may lead to deterioration in their condition, a risk that should be minimized, especially if a person is going through a crisis. Additionally, other users develop an overdependency on the AI tool, and interventions should be planned in a manner that would mitigate the risk of a long-term dependency. It is possible that AI-assisted communication, including with a therapist, could help deal with both of these risks.

We bring this population as an example, but these considerations pose open-ended questions that warrant exploration in the burgeoning domain of AI-mediated therapy broadly, where the interplay between human touch and technological aid is continually redefined.

## Patient Perceptions of AI Bots

In the growing literature examining individuals’ perceptions of AI bots, different factors have been shown to influence the levels of trust and bonding created with AI [35-38]. Much of this literature can be viewed from the same 2 axes: how helpful and capable the bot is in providing appropriate tools and results, and how empathic it is (in terms of understanding the user’s needs). One study showed that a therapeutic bot was rated on the dimensions of alliance at similar levels to face-to-face therapists [39]. In another study, responses generated by ChatGPT were rated as more authentic, professional, and practical [40]; however, participants were blind to the fact that responses were generated by conversational AI. When participants are aware of AI systems’ involvement or simply believe it is involved, responses can seem less authentic and less trustworthy and raise negative emotions [41-43]. Such findings require further research to determine whether responses can truly be experienced as empathic when one knows their artificial origin and whether such experiences differ in their relationship to treatment outcomes according to patients’ theory of change.

## Conclusions

The advent of advanced AI technologies offers substantial benefits and potential enhancements to therapeutic practices, as well as greater accessibility for a wider population. Nevertheless, certain junctures within the therapeutic process may be particularly sensitive to the need for human rapport. We suggest that those points, which may be whole treatments or specific sessions, are times when empathy is especially needed. Although conversational AI can adeptly simulate empathic interactions, sometimes creating the impression of empathy surpassing human capability [40], the essence of seeking empathy transcends the mere reception of an ideal empathic response. It encompasses a longing for the genuine care and emotional engagement of the individual offering support. The optimal path forward may lie in designing applications that facilitate therapist-AI partnerships, wherein AI systems could augment various facets of therapy—from initial intake and evaluation to, in certain instances, complete treatment modalities—while also consciously addressing the need for authentic human empathy, compassion, and care, when relevant for treatment success. However, most of our proposal is theoretical, and ultimately, we raise an empirical question that should be evaluated in future studies. We also encourage industry professionals developing AI applications for mental health and those conducting research within this domain to remain mindful of these considerations.

## Abbreviations

AI: artificial intelligence
IBI: internet-based intervention
